# *Curcuma aromatica* Water Extract Attenuates Ethanol-Induced Gastritis via Enhancement of Antioxidant Status

**DOI:** 10.1155/2015/582496

**Published:** 2015-09-21

**Authors:** Woo-Young Jeon, Mee-Young Lee, In-Sik Shin, Seong Eun Jin, Hyekyung Ha

**Affiliations:** ^1^K-Herb Research Center, Korea Institute of Oriental Medicine, 483 Expo-ro, Yuseong-gu, Daejeon 305-811, Republic of Korea; ^2^College of Veterinary Medicine, Chonnam National University, 77 Yongbong-ro, Buk-gu, Gwangju 500-757, Republic of Korea

## Abstract

*Curcuma aromatica* is an herbal medicine and traditionally used for the treatment of various diseases in Asia. We investigated the effects of* C. aromatica* water extract (CAW) in the stomach of rats with ethanol-induced gastritis. Gastritis was induced in rats by intragastric administration of 5 mL/kg body weight of absolute ethanol. The CAW groups were given 250 or 500 mg of extract/kg 2 h before administration of ethanol, respectively. To determine the antioxidant effects of CAW, we determined the level of lipid peroxidation, the level of reduced glutathione (GSH), the activities of catalase, degree of inflammation, and mucus production in the stomach. CAW reduced ethanol-induced inflammation and loss of epithelial cells and increased the mucus production in the stomach. CAW reduced the increase in lipid peroxidation associated with ethanol-induced gastritis (250 and 500 mg/kg, *p* < 0.01, resp.) and increased mucosal GSH content (500 mg/kg, *p* < 0.01) and the activity of catalase (250 and 500 mg/kg, *p* < 0.01, resp.). CAW increased the production of prostaglandin E_2_. These findings suggest that CAW protects against ethanol-induced gastric mucosa injury by increasing antioxidant status. We suggest that CAW could be developed for the treatment of gastritis induced by alcohol.

## 1. Introduction

Gastritis is inflammation of the gastric mucosa, which is essentially diagnosed by histology [1]. Excessive alcohol consumption causes acute hemorrhagic lesions, mucosal edema, epithelial exfoliation, and inflammatory cell infiltration, resulting in ulcers in the stomachs of humans and animals [2, 3]. Therefore, an animal model of ethanol consumption is widely used to assess the protective and healing activity of drugs in ulcer studies [4]. Oxidative stress is also involved in the gastric ulcerations caused by ethanol, nonsteroidal anti-inflammatory drugs, and cold-restraint stress [5, 6]. To discover potential anti-inflammatory therapeutic agents for gastritis, the effects of oxidative stress [7, 8] and decreased prostaglandin level [9] on gastric lesions have been studied extensively.

*Curcuma aromatica*, a perennial herb, belongs to the Zingiberaceae family [10].* C. aromatica* has been used for cosmetic formulations and traditional medicinal applications [11, 12].* C. aromatica* is used as an anti-inflammatory agent, to promote blood circulation, to alleviate blood stasis, and for the treatment of cancer [13]. Based on its properties, we hypothesized that* C. aromatica* may protect the stomach from gastritis because of ethanol consumption by enhancing the antioxidant system. The aim of this study was to evaluate the efficacy of* C. aromatica* water extract (CAW) for the relief of gastritis because of absolute ethanol-induced oxidative stress and destruction of the gastric mucosa and improvement in histological appearance of absolute ethanol-induced gastritis in rats.

## 2. Materials and Methods

### 2.1. Preparation of CAW

CAW was prepared in our laboratory from chopped* C. aromatica.* The extraction and high performance liquid chromatography analysis have been described previously [14].

### 2.2. Ethanol-Induced Gastritis

Sprague Dawley male rats (specific pathogen-free), weighing 200–250 g (6 weeks old), were purchased from Daehan Biolink Co. (Chungbuk, Korea) and used after one week of quarantine and acclimatization. The animal housing was as described previously [15]. This study was approved by the Korea Institute of Oriental Medicine (Daejeon, Republic of Korea) and was conducted according to the guidelines of the Institutional Animal Care and Use Committee. All experimental procedures were performed in compliance with the National Institutes of Health Guidelines for the care and use of laboratory animals and the National Animal Welfare Law of Korea.

Gastritis was induced by intragastric administration of absolute ethanol according to a method described previously [16–18], but with minor modifications. Thirty-five rats were divided into five groups and fasted for 18 h before the experiment. Rats in the control group were given phosphate-buffered saline (PBS) orally (5 mL/kg body weight), and the absolute ethanol administration group (EtOH group) received only absolute ethanol (5 mL/kg body weight) by oral gavage. Rats in a positive control group were given cimetidine (100 mg/kg body weight) by oral administration 2 h before the administration of absolute ethanol for three consecutive days. The fourth and fifth groups received CAW (250 or 500 mg/kg body weight, resp.) 2 h before absolute ethanol for 3 consecutive days. Cimetidine was used as a positive control because it has anti-inflammatory and antioxidant activities and has been widely used for the treatment of gastritis [19].

On the fourth day, the animals were sacrificed with an overdose of 100 mg/kg pentobarbital 24 h after treatment with the final ethanol administration. The stomach was removed, opened along the greater curvature, and rinsed gently in PBS and was stored at −70°C for later biochemical analysis.

### 2.3. Biochemical Analysis

Biochemical analysis was performed as described previously [15, 18, 20]. In brief, the stomach tissue was cut into small pieces and homogenized (1/10 w/v) with tissue lysis/extraction reagent and a protease inhibitor (Sigma, St. Louis, MO, USA). The homogenates were centrifuged at 12,000 rpm for 10 min at 4°C to precipitate cell debris, and the supernatant was used to measure levels of malondialdehyde (MDA), glutathione (GSH), catalase, and glutathione-S-transferase (GST). Total protein was determined using a protein assay reagent (Bio-Rad Laboratories, Hercules, CA, USA).

Lipid peroxidation was estimated by determining MDA using a thiobarbituric acid-reactive substances (TBARS) assay kit (BioAssay Systems, Hayward, CA, USA). GSH content was measured using a GSH assay kit (Cayman Chemical, Ann Arbor, MI, USA) and the results were expressed as *μ*mol/mg protein. The activities of antioxidative enzymes, including catalase, and GST were quantified using a commercial kit (Cayman Chemical) according to the manufacturer's protocols.

### 2.4. Measurement of Prostaglandin E_2_ (PGE_2_)

Biochemical analysis was performed as described previously [18]. In brief, the production of PGE_2_ was determined in a homogenate of gastric tissue using an enzyme-linked immunosorbent assay (ELISA) kit (Cayman Chemical), according to the manufacturer's instructions.

### 2.5. Histology

The stomach samples were preserved in 10% buffered formalin and processed for paraffin wax-blocks. Stomach tissues were embedded in paraffin wax, sectioned at 4 *μ*m thickness, and stained with hematoxylin (Sigma MHS-16) and eosin (Sigma HT110-1-32) solution to estimate inflammation and periodic acid-Schiff reagent (PAS; IMEB, San Marcos, CA, USA) to estimate mucus production.

### 2.6. Statistical Analysis

Data are expressed as means ± standard deviation. Statistical significance was determined using analysis of variance (ANOVA). If a test showed a significant difference between groups, the data were analyzed by a multiple comparison procedure using Dunnett's test. Statistical analysis was performed using SYSTAT version 10. The levels of significance were set as *p* < 0.05 and *p* < 0.01.

## 3. Results

### 3.1. Effects of CAW on Lipid Peroxidation and GSH in the Stomach of Rats with Ethanol-Induced Gastritis

**Figure 1:**
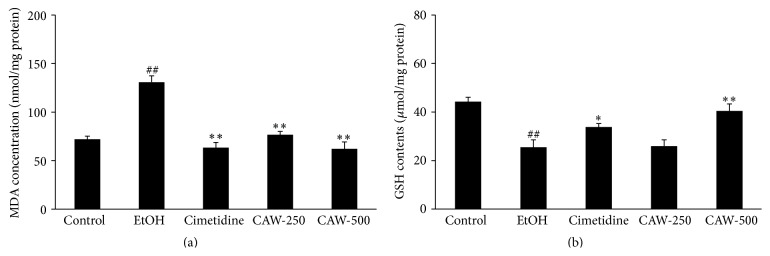
Effects of CAW on gastric MDA concentration (a) and GSH contents (b) in rats with absolute ethanol-induced gastritis. Each bar represents the mean ± SD of five rats. Control, normal control group; EtOH, absolute ethanol treatment group; cimetidine, ethanol + cimetidine (100 mg/kg); CAW-250, ethanol + CAW (250 mg/kg); CAW-500, ethanol + CAW (500 mg/kg). ^##^ *p* < 0.01 compared with the control group and ^*∗*^ *p* < 0.05 and ^*∗∗*^ *p* < 0.01 compared with rats in the EtOH group.

The concentration of MDA, an end product of lipid peroxidation, was greater in the stomach of rats in the EtOH group (130.75 ± 17.25 nmol/mg protein, *p* < 0.01) than in the stomach of control group (72.05 ± 8.39 nmol/mg protein) (Figure 1(a)). The MDA level in rats in the CAW-treated group was significantly reduced at 250 (76.60 ± 9.33 nmol/mg protein, *p* < 0.01) and 500 (62.11 ± 19.01 nmol/mg protein, *p* < 0.01) mg/kg compared with rats in the EtOH group. A significant reduction of MDA was observed in rats in the cimetidine-treated group (63.42 ± 14.11 nmol/mg protein, *p* < 0.01) compared with rat in the EtOH group.

**Figure 2:**
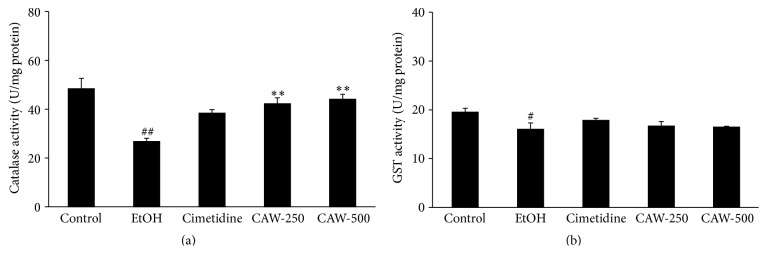
Effects of CAW treatment of rats with absolute ethanol-induced gastritis on gastric antioxidant enzymes including catalase (a) and GST (b). Control, normal control group; EtOH, absolute ethanol treatment group; cimetidine, ethanol + cimetidine (100 mg/kg); CAW-250, ethanol + CAW (250 mg/kg); CAW-500, ethanol + CAW (500 mg/kg). ^#^ *p* < 0.05 and ^##^ *p* < 0.01 compared with the control group and ^*∗∗*^ *p* < 0.01 compared with rats in the EtOH group.

By contrast with MDA, GSH contents in the stomachs of rats from the EtOH group (25.49 ± 7.50 *μ*mol/mg protein, *p* < 0.01) were significantly lower than that in the stomachs of rats from the control group (44.32 ± 4.01 *μ*mol/mg protein), and that in the stomachs of rats in the CAW group was only higher than that in the stomachs of rats in the EtOH group at 500 mg/kg (40.44 ± 6.52 *μ*mol/mg protein, *p* < 0.01) (Figure 2(b)). The GSH contents in the stomachs of rats from the cimetidine-treated group (33.80 ± 3.18 *μ*mol/mg protein, *p* < 0.05) were significantly higher compared with that in the stomachs of rats in the EtOH group.

### 3.2. Effects of CAW on Antioxidant Enzymes in Ethanol-Induced Gastritis

As shown in Figure 2(a), catalase activity in the stomach of rats in the EtOH group (26.78 ± 2.93 U/mg protein, *p* < 0.01) was less than that in the control group (48.39 ± 9.52 U/mg protein). However, CAW treatment resulted in a dose-dependent significant increase in catalase activity in the stomach of rats administered 250 (42.23 ± 5.51 U/mg protein, *p* < 0.01) and 500 (44.09 ± 4.43 U/mg protein, *p* < 0.01) mg/kg compared with rats in the EtOH group. However, the activity in the stomach of rats in the cimetidine-treated group (38.39 ± 3.61 U/mg protein) was higher compared to the activity in rats in the EtOH group, but the difference was not significant. As shown in Figure 2(b), the activity of other antioxidant enzymes, such as GST, was increased in the stomach of rats treated with CAW at 250 (16.70 ± 2.01 U/mg protein) and 500 (16.47 ± 0.39 U/mg protein) mg/kg compared with that in rats from the EtOH group (16.03 ± 2.85 U/mg protein), but the difference was not significant. The activity in the stomach of rats from the cimetidine-treated group (17.87 ± 0.87 U/mg protein) was greater than that in rats from the EtOH group, but the difference was not significant (Figure 2(b)).

### 3.3. Effects of CAW on the Production of PGE_2_ and Mucus

**Figure 3:**
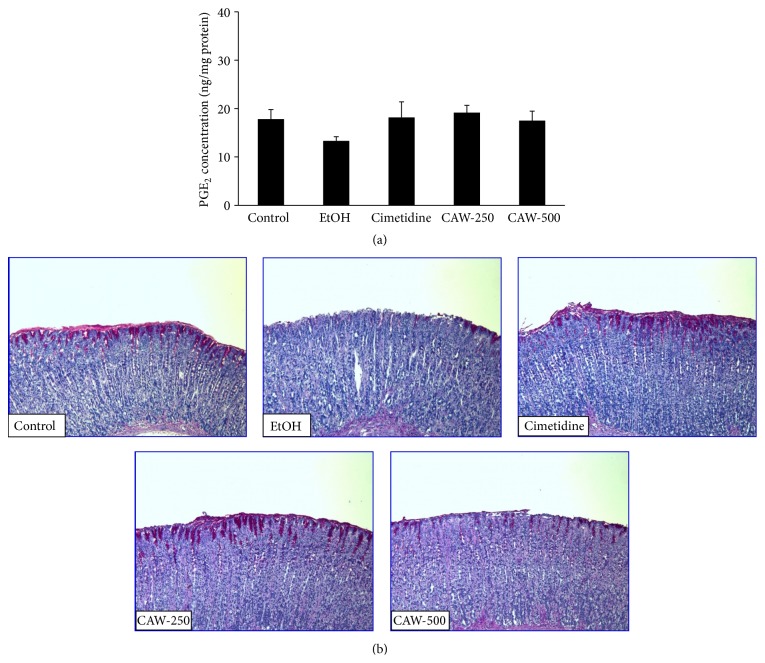
Effects of CAW on the production of PGE_2_ (a) and mucus (b) in the stomach of rats with absolute ethanol-induced gastritis. Histological sections were stained with periodic acid-Schiff (PAS, 50x). Control, normal control group; EtOH, absolute ethanol treatment group; cimetidine, ethanol + cimetidine (100 mg/kg); CAW-250, ethanol + CAW (250 mg/kg); CAW-500, ethanol + CAW (500 mg/kg).

As shown in Figure 3(a), the production of PGE_2_ was lower in the stomach of rats from the EtOH group (13.30 ± 1.96 ng/mg protein) than in the control group (17.81 ± 4.43 ng/mg protein). The production of PGE_2_ was strongly increased in rats treated with CAW at 250 (19.14 ± 3.99 ng/mg protein) and 500 (17.51 ± 5.21 ng/mg protein) mg/kg compared with rats in the EtOH group. The production of PGE_2_ in the stomach of rats in the cimetidine group (18.18 ± 8.54 ng/mg protein) increased compared with that in rats in the EtOH group, but the difference was not significant. The PAS staining of mucus in stomachs from rats in the EtOH group was weaker than that observed in stomachs from control group. Mucus staining was more intense in stomachs from rats in CAW- and cimetidine-treated groups (Figure 3(b)).

### 3.4. Effects of CAW on Inflammation in Stomach

**Figure 4:**
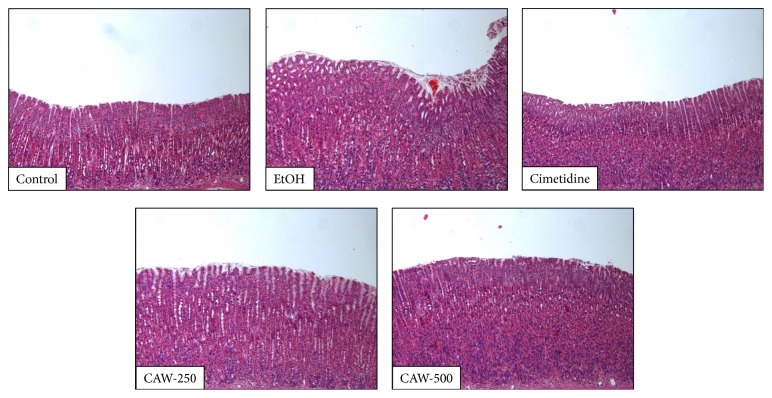
Effects of CAW on inflammation in the stomach of rats with absolute ethanol-induced gastritis. Histological sections were stained with hematoxylin and eosin (H&E, 50x). Control, normal control group; EtOH, absolute ethanol treatment group; cimetidine, ethanol + cimetidine (100 mg/kg); CAW-250, ethanol + CAW (250 mg/kg); CAW-500, ethanol + CAW (500 mg/kg).

Administration of absolute ethanol caused hemorrhagic injury and inflammation in stomach. In the EtOH group, extensive inflammatory cell infiltration in the mucosa and submucosa area was observed. However, CAW groups attenuated the degree of inflammation than in the EtOH group (Figure 4).

## 4. Discussion

Gastroprotective factors include mucus, prostaglandins, mucosal antioxidants, and gastric blood flow. It is well known that alcohol, stress, and inflammatory drugs increase gastric injury including hemorrhage, erosion, and ulceration [21]. In particular, exposure of the mucosa to ethanol results in damage to the mucosal membranes [22, 23]. Usually, ethanol can rapidly penetrate the gastric mucosa because it is able to solubilize the protective mucus. In the present study, our findings are consistent with features of ethanol-induced gastric damage described in the literature. Administration of CAW reduced acute gastritis by decreasing MDA levels and increasing the components of the antioxidant system including GSH and catalase. Production of PGE_2_, associated with mucus production, was increased.

The peroxidation of polyunsaturated fatty acids and the subsequent formation of free radicals may be involved in the pathogenesis of gastritis [24]. It is well known that tissues exposed to oxidative stress contain large amounts of toxic oxygen radicals, which induce lipid peroxidation that produces MDA [25, 26]. MDA levels increase in gastric tissue treated with absolute ethanol [15, 18, 20]. In the present study, as an indicator of lipid peroxidation, the MDA levels significantly increased in the stomach of rats in the EtOH group compared with control group. However, a significantly reduced MDA level was found in the stomachs of rats in the CAW group compared with rats in the EtOH group. The reduced level of MDA content in stomachs from rats in the CAW group suggests that CAW has a protective action on gastric mucosa by decreasing lipid peroxidation.

Production of oxygen free radicals may play a crucial role in the development of ethanol-induced gastric lesions [22, 27]. The antioxidant activity of GSH, catalase, glutathione peroxidase, glutathione reductase, superoxide dismutase, and GST may be involved in the defense system of the stomach against oxidative stress caused by ethanol [28, 29]. Depletion of gastric mucosal GSH may result in the accumulation of free radicals that can initiate membrane damage by lipid peroxidation. GSH is one of the most important agents in the antioxidant defense system [21], and a deficiency of GSH puts cells at risk of oxidative damage [30]. In the present study, a decreased level of GSH was found in the stomach of rats in the EtOH group compared with control group. These results are consistent with our previous findings that a decreased level of GSH can potentiate gastric injury induced by absolute ethanol intake, and enhancement of GSH levels shows gastroprotective effects [15, 18, 20]. CAW treatment increased the level of GSH in the stomachs of rats in a dose-dependent manner (although this was only significant at the highest dose) compared with the levels found in the stomachs of untreated rats from the EtOH group.

Oxidative stress and depletion of antioxidants have been considered a primary key step in alcohol-induced gastritis and have been investigated widely [31]. Catalase catalyzes the decomposition of hydrogen peroxide to water and oxygen, which protects cells and tissues against oxidative damage [32]. Catalase may provide defense against oxidative damage to the gastric mucosa after administration of ethanol [33]. GST, another antioxidant enzyme, catalyzes the conjugation of GSH to electrophilic centers on a wide variety of substrates via a sulfhydryl group [21]. In our present study, the activities of these enzymes were increased in the stomach after CAW treatment of rats administered ethanol, which suggests that an increase of these enzymatic activities was, at least in part, responsible for reducing oxidative tissue damage to the stomach after ethanol intake. Therefore, these findings suggest that CAW protects the stomach against the gastritis caused by a decrease in the activities of free-radical scavenging enzymes such as catalase and GST in the stomach tissue.

Impaired microcirculation is a reason for gastric mucosal barrier damage and is accompanied by declining levels of PGE_2_ in the blood and gastric mucosa [34, 35]. PGE_2_ accelerates the flow of the gastric mucosal microcirculation, promotes the secretion of bicarbonate, mediates the adaptive immune protective function, increases protein synthesis and cell renewal, and enhances the ability of damaged gastric mucosa to repair itself [36]. Consistent with the findings of previous studies, the present study showed that CAW treatment elevates gastric PGE_2_ content.

## 5. Conclusions

CAW showed a protective effect on ethanol-induced gastritis and may be a candidate for gastritis therapy because it inhibits lipid peroxidation and increases levels of GSH, catalase, GST, and PGE_2_.
